# Diagnosis and Treatment of Infantile Hemangioma from the Primary Care Paediatricians to the Specialist: A Narrative Review

**DOI:** 10.3390/children11111397

**Published:** 2024-11-18

**Authors:** Francesco Bellinato, Maria Marocchi, Luca Pecoraro, Marco Zaffanello, Micol Del Giglio, Giampiero Girolomoni, Giorgio Piacentini, Erika Rigotti

**Affiliations:** 1 Department of Medicine, Section of Dermatology, University of Verona, 37126 Verona, Italy; francesco.bellinato@univr.it (F.B.); giampiero.girolomoni@univr.it (G.G.); 2 Pediatric Unit, Department of Surgical Sciences, Destiny, Gynecology and Pediatrics, University of Verona, 37126 Verona, Italy

**Keywords:** children, infants, infantile haemangioma, propranolol

## Abstract

Infantile haemangiomas (IHs) affect 3–10% of infants, 10% of whom need topical or systemic beta-blocker therapy. Propranolol is the first choice for IHs with a high risk of complications. Since more than half of IHs leave a permanent mark, to reduce outcomes, it is essential to start oral propranolol (2–3 mg/kg/day in 2 doses/day) within the 5th month of life (i.e., during the proliferative phase) and to complete the therapy cycle for at least 6 months. This review aims to summarise the epidemiology, clinical presentation, diagnosis, and treatment of IHs and to highlight the importance of proper referral to specialised hub centres. Patients with vascular anomalies, particularly those suspected of having IH, should be referred to a specialised centre for accurate diagnosis, management by a multidisciplinary team, and timely treatment. IHs may pose life-threatening, functional, and aesthetic risks or may ulcerate. Segmental infantile haemangioma of the face/neck and the lumbosacral regions can be associated with various malformations. To ensure timely specialist evaluation and treatment to reduce the potential risk of complications, it is essential to identify high-risk IHs rapidly. The Infantile Haemangioma Referral Score (IHReS) scale is an important tool to assist primary care paediatricians and general dermatologists.

## 1. Epidemiology

Infantile haemangiomas (IHs) are the most common vascular tumour in paediatric patients, with a female predominance (2.3–2.9 times higher). IHs affect approximately 4–5% of infants, with incidence rates reported to range from 2% to 10% [1,2,3,4]. Such variability may be due to incomplete registry data, as IHs often become apparent only after infants are discharged from the hospital. For example, an Australian study reported an incidence of 2.6%, based on parent-reported cases and follow-ups until 6 weeks of age. A U.S. study found a 4.5% incidence in 594 infants up to 9 months, though it identified only 27 cases and relied on phone follow-up. A recent large Dutch study reported a 9.9% prevalence in children aged 0 to 16 months. Additional challenges in determining the true incidence include nonattendance at follow-up medical appointments, referral biases, and inconsistencies in the terminology of vascular anomalies, with a standardised classification only established in 1982 [5]. However, as early as 1955, Holmdahl et al. reported that “it is difficult to study the literature because of the varying nomenclature” [6]. Despite these challenges, there has been a significant and consistent increase in the incidence of IHs over the past four decades. This rise is potentially linked to an increase in premature births and low birth weights.

## 2. Risk Factors

Different epidemiological studies have identified certain maternal and perinatal factors as risk factors for the development of infantile haemangiomas (IHs). According to a recent large matched case-control study by Gong X et al., independent risk factors for IHs are: miscarriage history, anaemia in pregnancy (OR = 4.2; 95% CI [3.1–5.8]), preterm premature rupture of membranes (PPROM) (OR = 3.2; 95% CI [1.4–7.5]), placenta previa (OR = 2.4; 95% CI [1.8–3.3]), threatened miscarriage (OR = 2.3; 95% CI [1.7–3.0]), premature rupture of membranes (PROM) (OR = 1.8; *p* < 0.05), progesterone use (OR = 1.6; *p* < 0.001), and abnormal amniotic fluid volume (OR = 1.5; *p* < 0.05). Conversely, gestational diabetes mellitus (OR = 0.6; 95% CI [0.5–0.8]), multiple gestations (OR = 0.4; 95% CI [0.2–0.7]), hypothyroidism (OR = 0.4; 95% CI [0.2–0.7]), and uterine fibroids (OR = 0.4; 95% CI [0.3–0.6]) are associated with a reduced risk of IHs [7].

## 3. Classifications of IH

IHs are classified based on anatomical and clinical characteristics into superficial, deep, and mixed IH [8,9]. A superficial IH presents as papular or nodular lesions with a red or reddish-purple colour, featuring a smooth or lobulated surface and a tense, elastic consistency. The base is rarely pedunculated. Sizes can range from a few millimetres to a large area, such as an entire limb or half of the trunk. A deep IH manifests as a well-defined, elastic nodular swelling, typically covered by normal or bluish skin, or sometimes a telangiectatic hue. A mixed IH combines features of both superficial and deep types.

Based on their distribution, IHs can also be categorised as focal, multifocal, segmental, and indeterminate [10]. Focal IHs appear as well-defined, localised lesions. Multifocal IHs are characterised by the presence of multiple haemangiomas in different locations. Segmental IHs present as a lesion affecting a more or less extensive anatomical region, often resembling a plaque and usually larger than 5 cm in diameter. Finally, indeterminate IHs are neither localised nor segmental; they are also called “partially segmental”. Additionally, IHs can be extra-cutaneous, affecting organs such as the liver, parotid gland, and upper airways.

Distinct clinical features characterise IH with minimal or arrested growth (IH-MAG). It typically appears as a pink, blotchy, or reticulated patch on the skin, often with areas of vasoconstriction or a surrounding blanching halo. Fine or coarse telangiectasia (small, visible blood vessels) may be present. During the proliferative phase, bright red papules can develop, particularly around the edges of the lesion. IH-MAG has been associated with atrophic, hypertrophic, and eczematous dermatitis [11].

## 4. Etiopathogenesis

Different studies have been unable to identify a specific gene responsible for the formation of IH. While a genetic mechanism is likely significant, it is highly improbable that it alone drives IH pathogenesis. Several genes involved in angiogenesis, cell growth, transcriptional control, and cell signalling are deregulated in IHs, and the expression of different genes is also altered by beta-blocker treatment. In particular, 6-phosphofructo-2-kinase 3 (PFKFB3), Angiopoietin 2 (ANGPT2), Vascular endothelial growth factors (VEGF, VEGFA, VEGF2), Glucose transporter-1 (GLUT1), Stromal cell-derived factor-1a (SDF1α), Matrix metalloproteinase 9 (MMP9), and many others have been found upregulated. Conversely, KiSS1 metastasis suppressor (KISS1), Forkhead Box O1 (FOXO1), and Cyclin-dependent kinase inhibitor 2A (CDKN2A or p16) have been found to be downregulated in all phases, which also applies to Collagen type IV alpha 2 chain (COL4A2), Integrin subunit alpha 1 (ITGA1), Platelet-derived growth factor receptor-beta (PDGFRB), RNA binding Fox-1 homolog 2 (RBFOX2), Matrix-remodeling-associated protein 5 (MXRA5), Vasohibin 1 (VASH1), and Peroxidasin (PXDN) but specifically in the involution phase [12].

An IH results from the dysregulation of both vasculogenesis and angiogenesis, though the exact triggers for its development remain debated. No single hypothesis thoroughly explains all the characteristics of IHs. The literature reports that the clonal proliferation of endothelial cells, leading to vasculogenesis, is likely triggered by a hypoxic stimulus [13]. Progenitor endothelial cells migrate to sites with favourable growth conditions, stimulating angiogenesis by releasing noradrenaline from sympathetic nerve endings and endothelial cells. These cells, which produce catecholamines under normal conditions, increase their expression under hypoxic conditions. Noradrenaline binds to beta2- and beta3-adrenergic receptors on endothelial cells, activating intracellular pathways that lead to the overproduction of vascular endothelial growth factor (VEGF) via the hypoxic-inducible factor alpha (HIF-alpha) pathway. Noradrenaline also stimulates the release of stromal cell-derived factor-1α (SDF1-alpha), which mobilises stem cells with high proliferative potential, enabling their proliferation and differentiation into immature endothelial cells, dendritic cells, and mesenchymal cells with adipogenic potential, thereby laying the groundwork for haemangioma formation.

Recent evidence suggests that the renin–angiotensin system (RAS) plays a significant role in developing an IH, with the vasoactive peptide angiotensin II (ATII) acting as a critical regulator of hemogenic endothelium. Studies have shown the expression of ATII receptor 2 (but not ATII receptor 1) in endothelial progenitor cells during the proliferative phase of an IH. It is hypothesised that ATII stimulates the secretion of VEGF and osteoprotegerin, which promote vasculogenesis and protect against apoptosis [14,15].

The increased expression of HIF-alpha also enhances glucose transporter type (GLUT1) expression on endothelial cells, distinguishing IHs from other vascular malformations. Missense mutations in vascular endothelial growth factor receptor 2 (VEGFR2) and variants of ring finger protein 213 (RNF213), associated with PHACE syndrome, are additional factors, along with placental abnormalities (such as placenta previa and preeclampsia), disruptions in placental circulation, advanced maternal age, and prematurity. Recent studies suggest aquaporin, a membrane protein expressed by telocytes (stem cells surrounding the haemangioma’s vessels, outside of endothelial cells and pericytes), which facilitates water transport into cells, is overexpressed in haemangioma endothelial cells compared to normal vascular cells. A combination of bevacizumab and propranolol reduces this overexpression. In light of these new pathophysiological insights (involving hypoxia, catecholamines, aquaporin, and telocytes), an IH may be described as a “blood-skin barrier adrenergic tumour” [16,17].

## 5. Natural History

Haemangiomas are rarely present at birth but more commonly appear within the first few weeks of life. Sometimes, a precursor lesion may manifest as pallor, a pale bluish spot, or an area with fine telangiectasias. The lifecycle of an IH is divided into three phases [18,19]:

1. *Rapid Proliferative Phase* (0–1 year):
  - *Early proliferative phase* characterised by rapid growth. It occurs within the first 3–5 months, with the fastest growth velocity typically seen between weeks 5 and 8. By the end of this phase, IHs reach about 80% of their final size.
  - *Late proliferative phase*, which is slower, usually completing by 9–12 months of age, though growth may continue up to 36 months, marking late growth.
  Only 3% of IHs continue to grow beyond 9 months. Generally, superficial forms grow until the 5th month, while deep and segmental forms appear later and continue to grow for a more extended period (up to 18 months) [20]. Superficial IHs are more prone to residual scarring than deep ones, significantly if the lesion lightens within the first 3 months, which can be an early sign of ulceration.
2. *Involution Phase* (1–5 years): This period is characterised by a softening of the lesion and fading of its colour, starting from the centre, with a progressive reduction in volume and decreased vascularisation.
3. *Involuted Phase* (5–10 years): In this phase, complete or near-complete regression occurs, sometimes leaving residual scarring such as loose skin, atrophy, telangiectasias, and/or fibro-fatty tissue [21].

An infantile haemangioma with minimal or absent growth (IH-MAG) represents an uncommon variant, marked by limited or no proliferation. Unlike typical IHs, around three out of four of these lesions are visible at birth. If a proliferative phase does occur, it usually affects only part of the lesion, most often at the periphery. IH-MAGs follow a similar involution process to other IHs [9].

## 6. Diagnosis

IHs are generally diagnosed clinically. Imaging studies (i.e., ultrasound (US) and MRI) and other investigations (i.e., colorimetry) are required for special situations. High-frequency US and Doppler can be used in the case of deep IHs, multifocal or hepatic IHs, segmental IHs, and midline IHs of the lumbosacral area and in the differential diagnosis of vascular anomalies. The purpose of US is to evaluate the depth and size of the lesions, check for intrahepatic lesions, and rule out renal and urogenital anomalies and spinal dysraphism. The US characteristics of an IH vary according to their stage in the natural progression. During the proliferative phase, high-frequency US reveals a well-defined, hypoechoic, and hypervascular tumour with rapid blood flow. In contrast, in the involution phase, the tumour decreases in size, becomes more echogenic, and shows a reduction in both the density and the size of blood vessels [9]. MRI is the most useful imaging technique for helping to define the extent and tissue characteristics of vascular tumours. MRI for segmental IHs is helpful to rule out intracranial, cerebrovascular, or spinal anomalies. Contrast can help to differentiate IHs from other tumours. Echocardiography can be used in large or multifocal IH, PHACE syndrome, and lumbosacral IHs to rule out cardiac insufficiency and cardiac or aortic anomaly. Increased vascularity on pre-treatment US was found to be significantly associated with successful treatment for propranolol, whereas prominent fat component on MRI was significantly associated with treatment failure [22].

IHs can occur anywhere on the body but most commonly affect the head and neck, particularly over bony prominences (such as the central facial area). Differential diagnosis can be challenging, particularly for deep IHs in specific locations. Differential diagnosis includes other vascular tumours, such as congenital haemangioma, tufted angioma, kaposiform haemangioendothelioma, pyogenic granuloma, and vascular malformations.

Typically, a vascular malformation is present from birth, grows minimally or very slowly over many years, and does not undergo spontaneous regression. IH precursors and early proliferating lesions may sometimes be misdiagnosed as capillary malformations or telangiectasia. For infants with growing tumours, if the diagnosis of an IH is not clinically evident, a biopsy should be performed.

Histopathologically, IHs in the proliferative phase consist of lobular clusters of capillaries lined by plump, mitotically active endothelial cells surrounded by pericytes. As IHs enter the involution phase, the number of vessels decreases, the endothelium becomes flatter and less mitotically active, and apoptotic debris appears within a more prominent fibrous and fatty stroma. GLUT-1 staining on the endothelium serves as a reliable marker for an IH and is typically absent in other vascular tumours and malformations [9].

Segmental lesions are clinically and prognostically significant as they may be associated with underlying anomalies. For instance, extensive facial haemangiomas may be linked with PHACE syndrome, while segmental lesions affecting the anogenital or lumbosacral midline can be associated with PELVIS, LUMBAR, and SACRAL syndromes. PHACES Syndrome (OMIM 606519) is an acronym for a group of abnormalities associated with large, segmental haemangiomas, especially on the face. It includes posterior fossa brain malformations, haemangiomas (usually large facial haemangiomas), arterial anomalies (especially involving the head and neck), cardiac defects (commonly a coarctation of the aorta), and eye abnormalities. Sternal or midline defects and hamartomas are also often associated with this syndrome, and some refer to it as PHACES, including an S for sternal defects/supraumbilical raphe. LUMBAR syndrome involves haemangiomas of the lower body and possible associated defects: lower body haemangiomas (often in a lumbosacral location), urogenital anomalies, myelopathy or spinal cord abnormalities, bony deformities, anorectal malformations, and renal anomalies. In LUMBAR, the most severe malformation is often a lipomyelomeningocele, necessitating an MR of the spinal cord and abdomen/pelvis in all neonates with segmental IHs in these regions, even if neurologically asymptomatic [9].

The occurrence of five or more IHs is uncommon, representing about 3% of cases. The term haemangiomatosis is applied when there are between five and several hundred small, multifocal IHs. The liver is the most frequently affected extracutaneous site, with the severity of hepatic haemangiomas ranging from asymptomatic to life-threatening, with fatalities primarily resulting from congestive heart failure. Hepatic haemangiomas can be focal, multifocal, or diffuse [23]. Hypothyroidism due to the expression of iodothyronine deiodinase was found to be associated with this condition. Children with diffuse hepatic IHs (as well as those with large cutaneous IHs) are at increased risk of developing high-output cardiac failure. The early identification of infants with cutaneous IHs who are at risk for internal haemangiomas is considered essential. Currently, screening for internal haemangiomas is recommended for infants who present with five or more cutaneous IHs. Other possible extra-cutaneous sites include the larynx (in segmental haemangiomas), the parotid gland, and, more rarely, the central nervous system (intracranial or intraspinal) [24].

**Figure 1:**
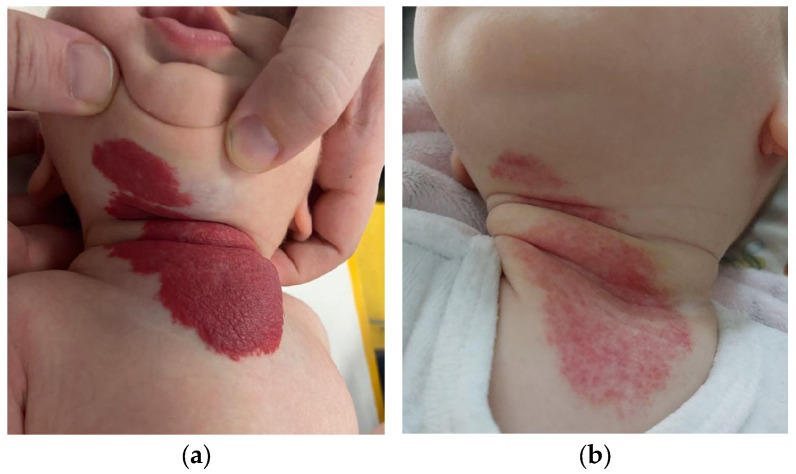
A 12-week-old female infant presented with a mixed IH extending to the neck, chin, and sternal region, requiring multidisciplinary management for treatment with propranolol, including ultrasound and ENT consultation with fiber optic laryngoscopy (**a**). Telangiectatic residuals were observed after three months of treatment with propranolol (**b**).

**Figure 2:**
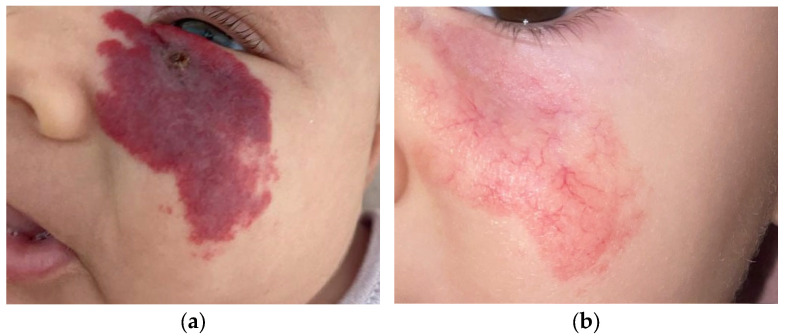
A 6-week-old male infant presented with an ulcerated IH on the left malar region, treated with propranolol for aesthetic risk and multispecialty diagnostics to rule out PHACE syndrome (including ophthalmologic, ENT and cardiologic consultations, echocardiogram, ECG, and brain MRI) (**a**). Telangiectatic residuals at 12 months of age (**b**).

To ensure timely specialist evaluation and reduce the potential risk of complications, it is essential to identify high-risk IHs, including:

(1) Life-threatening risks, such as obstructive subglottic haemangiomas and large haemangiomas causing cardiac or hepatic failure [25]. Obstructive subglottic haemangiomas can lead to significant airway obstruction (Figure 1), while large haemangiomas might contribute to cardiac failure or hepatic dysfunction due to high-output cardiac failure.
(2) Functional impact risks, such as periorbital haemangiomas, which may impede complete eye-opening, especially during the peak proliferation phase (2–3 months of life), potentially leading to permanent amblyopia, astigmatism, strabismus, proptosis, and optic nerve compression. Nasal, labial, or laryngotracheal haemangiomas can obstruct airways, posing life-threatening risk—haemangiomas on joints, which may limit the mobility of the affected segment, among other functional impairments.
(3) Aesthetic risks with psychological implications, as haemangiomas located on the face (Figure 2), including the glabella, nose, philtrum, chin, cheeks, and lips, which may result in permanent deformity; on the mammary gland in females; on the genitals in both males and females; and ulcerated haemangiomas that do not respond to local treatments, causing pain and subsequent permanent aesthetic damage.
(4) Ulcerations, the most common complication of haemangiomas, particularly between the 4th and 8th months of life, occurring in 10–25% of patients referred to specialised centres. The areas at highest risk of ulceration include the lips, head and neck region, skin folds, and buttocks. Clinically, approximately 50% of haemangiomas in the perineal region and 30% of those on the lower lip are at risk of ulceration.
(5) Structural anomaly risks, such as those seen in PHACES or LUMBAR syndromes [26,27,28,29].

For IHs at risk of complications, early referral to a specialised centre is recommended to enable timely intervention and improve therapeutic outcomes. Certain locations require a multi-speciality approach. Specifically, ophthalmologists should be involved for periorbital haemangiomas to address potential visual impairments and related complications. To manage potential airway obstruction or auditory issues, otolaryngologists should be consulted for laryngeal and auricular haemangiomas. Cardiologists should be consulted to identify cardiac anomalies associated with segmental haemangiomas and hepatic haemangiomas at risk of high-output cardiac failure. Neurosurgeons are essential for evaluating cerebral anomalies associated with PHACE syndrome.

To determine whether a patient should be referred to a specialised centre, paediatricians can use the Infantile Haemangioma Referral Score (IHReS), a validated assessment tool developed by an international group of experts and tested by paediatricians [30]. The IHReS aims to improve healthcare providers’ ability to decide if and when to refer patients with IHs to a specialised centre. The tool begins with identifying risk factors through simple YES/NO questions, including:

- Complications or potential risk of complications (ulcerations, visual deficits, feeding difficulties, laryngeal stridor).
- Location in the midface and/or ears, mammary region (in females), or lumbosacral midline.
- Size ≥ 4 cm (focal or segmental).
- Number of haemangiomas ≥ 5.

In case of a positive response, it is necessary to refer the patient. If all questions are answered with NO, a more detailed table should be completed with information on:

- the location of the haemangioma,
- the size of the most extensive haemangioma,
- the child’s current age,
- the growth of the haemangioma in the past two weeks.

If the score is 4 or higher, the patient should be referred to a specialised centre. If the score is less than 4, the primary care provider will monitor the patient and reassess the score at each follow-up visit during the first 6 months.

## 7. Treatment

Therapy is necessary in approximately 12% of cases, considering the potential for spontaneous involution. Treatment is indicated for ulcerated IHs, those that pose a life-threatening risk, cause functional impairment, or result in permanent aesthetic harm [31]. According to current guidelines, the therapeutic strategies for treating IHs include:

- *Topical Timolol*: Timolol maleate 0.5%, a nonselective beta-blocker, is a well-tolerated, safe, and effective treatment for thin superficial IHs. It is as effective as oral propranolol for treating superficial IHs, with fewer systemic adverse events and superior efficacy to topical corticosteroids. It should be applied directly to the lesion using a 0.5% eye drop solution at a dose of 1–2 drops twice daily or as a 0.5% timolol maleate gel (15-min applications, 3 times a day). Treatment should continue for 6–9 months [32,33].
- *Oral Propranolol*: First recognised as effective in 2008, oral propranolol is now considered the first-choice treatment for IHs at risk of complications. The effective dose ranges from 2 to 3 mg/kg body weight per day, with a treatment duration of at least 6 months, continuing until the child is 12–18 months old. Although the initiation of propranolol is recommended during the proliferative phase, it can still lead to improvement when started after 9–12 months of age. The mechanism of action of propranolol, beta-blocker, is yet to be elucidated; however, theories include vasoconstriction, the inhibition of angiogenesis, the induction of apoptosis, the inhibition of nitric oxide production, and the reduction of renin levels and consequently of the renin–angiotensin axis.

It is effective only for GLUT1-positive haemangiomas, such as IHs, and does not work on GLUT1-negative congenital haemangiomas. This therapy has proven effective and safe over the years. It should be initiated as early as possible, following cardiology clearance for selected cases, including a tolerance test for the child, dose titration, and parental education, particularly on preventing side effects. Propranolol treatment is contraindicated in cases of asthma, hypotension, pheochromocytoma, and certain heart conditions, such as second and third-degree atrioventricular block, sinus node disease, cardiogenic shock, bradycardia, and heart failure. Propranolol-resistant IHs were defined as failure to achieve the expected therapeutic response to propranolol after the oral administration of propranolol ≥ 2 mg/kg/day for at least 4 weeks. Propranolol-resistant IHs are uncommon, but they have been described in the literature with an estimated incidence ranging from 1 to 13%. Risk factors for recurrence reported in the literature are the discontinuation of treatment before 12 months of age, a mixed- or deep-type IH, female gender, multiple IHs, a segmental-type IH, and location on the nasal tip [34,35,36]. Oral atenolol, a hydrophilic β-1 receptor-selective beta-blocker, may represent a valid treatment alternative. Nonetheless, there is still controversy regarding the efficacy and safety of atenolol when compared with propranolol as monotherapy for this condition.

- *Steroids*: Considered a second-line treatment if there is no response to propranolol, but perhaps the first choice for patients with cardiovascular disease. The most commonly used drug is oral prednisolone, administered at a dose of 2–3 mg/kg/day for 12 weeks, followed by a gradual tapering until the child is 9–12 months old. Intralesional injections of triamcinolone and/or betamethasone (every 4–6 weeks) may be considered for bulky focal haemangiomas in the proliferative phase or in specific locations (e.g., parotid gland, lips) [37].
- Additional systemic treatment options include captopril, sirolimus, and intravenous vincristine; however, their use is limited in infants due to the risk of adverse reactions and side effects.
- *Laser* or *Traditional Surgery*: Residual lesions after propranolol therapy, usually telangiectatic, may require surgical or laser treatment at specialised centres with expertise in haemangioma surgery. Most studies report the use of pulsed dye laser or long-pulse Nd laser. The combination of laser with systemic beta-blockade has demonstrated superiority over monotherapy [24].

## 8. Operative Algorithm for Propranolol Therapy

Before treatment, a thorough medical history review and clinical examination should be performed to identify any potential contraindications. Routine echocardiography and ECG are unnecessary if the basic cardiology examination is normal. However, an ECG and cardiology consultation are required if bradycardia or arrhythmia is detected during auscultation. Treatment should be initiated only in clinical settings equipped and qualified to manage adverse events, such as bradycardia. Inpatient initiation should be considered for infants with a corrected age of less than 2 months, weight under 2 kg, inadequate social support, or comorbidities affecting the cardiovascular or respiratory system or blood glucose levels. Propranolol is administered at a starting dose of 1 mg/kg daily, divided into two doses during the first week. The dose gradually increases to 2–3 mg/kg daily over the following weeks. Patients should be monitored for 2 h after the first dose and at each increase. During the maintenance, propranolol should be continued at 2–3 mg/kg per day, divided into two doses, for 6 months. Monthly clinical evaluations and photographs should be carried out to monitor progress. Tapering off the medication is not necessary at the end of treatment. Finally, parents should be informed about the risk of relapse, which occurs in 10–15% of cases. Parents should also be informed about the risks of hypoglycaemia and respiratory symptoms at each visit, such as wheezing. If the infant experiences poor food intake or wheezing, propranolol should be stopped. However, the dosage should not be adjusted for minor side effects like cold hands or asymptomatic low diastolic blood pressure [29].

## 9. Hub and Spoke: Management of IH from Primary Care to Specialist Centres

The American Academy of Pediatrics has issued guidelines for managing IHs, as have working groups in the UK, Europe, and Australia. While these guidelines have some differences, they are mostly consistent, and the recommendations in this article align closely with them [30,38].

Patients with vascular anomalies, especially those suspected of having an IH, should be referred to a specialised centre for accurate differential diagnosis and timely treatment to avoid complications that may affect vital functions or cause permanent cosmetic damage [30]. It is crucial to address these patients early, ideally within the recommended age range of 5 weeks to 5 months, when treatment options are most effective [30]. Additionally, many patients diagnosed with an IH start off-label systemic propranolol therapy. Although this practice is generally safe, it may produce less satisfactory results. Systemic propranolol is administered in specialised hospital settings. Therefore, primary care paediatricians and outpatient dermatologists require training to ensure proper referral and management by a multidisciplinary team. The multidisciplinary team at the specialised centre can conduct diagnostic tests, including a comprehensive abdominal ultrasound to check for hepatic haemangiomas in cases of multiple lesions, a cardiological evaluation of present cardiovascular risk factors, and an MRI for haemangiomas associated with potential syndromic conditions. The primary care paediatrician can receive regular updates on the patient’s clinical status through reports from hospital visits and will have direct contact for urgent communications. To improve information dissemination and the referral process, the following methods can be implemented:

- *Informational Leaflet*: An informational flyer for primary care paediatricians summarises patient management by the multidisciplinary team, including the appropriate age for referral and key treatment indications. The leaflet also includes the IHReS scale to aid clinical decision making and outlines diagnostic tests and patient follow-up procedures.
- *Annual Courses/Meetings*: Organised through significant professional associations to update paediatricians on new guidelines and therapeutic approaches.
- *Electronic Medical Records Integration*: Adding a specific section in the database system used by primary care paediatricians. When the term “infantile haemangioma” is entered, an automatic link to the IHReS scale is provided, allowing paediatricians to complete, save, print, or email the form to the specialist centre.
- *Website Updates*: The emangioma.net website has been updated with information for both parents and clinicians, including a map of referral centres with contact details.

## 10. Direct Referral Pathway for Patients with IH to a Specialist Referral Centre

The primary care paediatrician and/or the outpatient dermatologist, when suspecting the presence of an IH but lacking specific knowledge on the subject, may use the IHReS scale as a diagnostic aid to determine if a patient with a suspected or confirmed IH should start treatment with propranolol [29]. The score is always available online at https://www.ihscoring.com/it (accessed on 16 November 2024). In cases of suspected vascular anomalies, particularly haemangiomas, or if the score is higher than 4, or if there is a “yes” response to one of the initial questions, the outpatient physician or a hospital physician not specialised in treating vascular anomalies should refer the patient to the specialised referral centre. The referral should include a request for an initial paediatric-dermatology consultation, where the lesion will be evaluated, and guidance will be provided on starting systemic treatment with propranolol or topical treatment with timolol. Appointments are made with a referral, and the primary care paediatrician may contact the centre’s referring paediatrician directly, sending photographic documentation and the IHReS scale result to assess the most appropriate timing for the patient’s care.

Three aspects that the primary care paediatrician or dermatologist should consider in the medical history are the age at haemangioma onset (as a haemangioma present at birth does not require systemic treatment with propranolol), complications that arose in the mother during pregnancy, and the prematurity of the newborn, as these are risk factors for the development of IHs.

**Figure 3:**
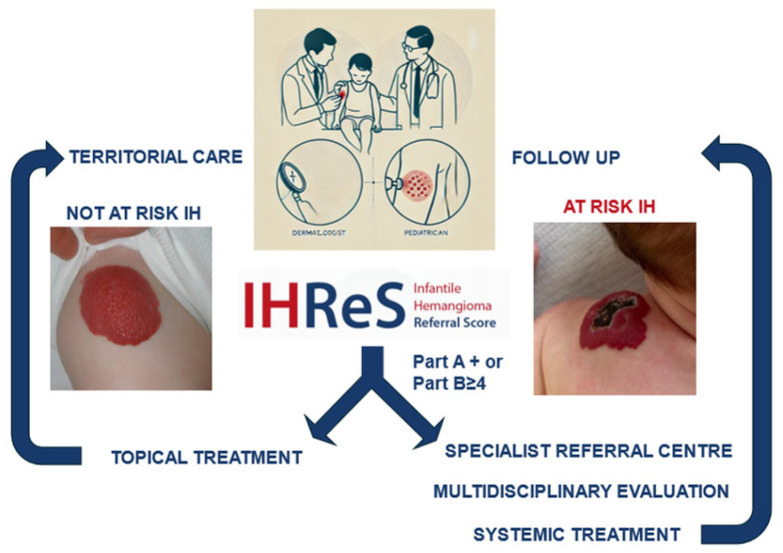
Referral pathways of IH from primary care to specialist centres.

For those starting systemic treatment with propranolol, an informational brochure can be provided for their primary care paediatrician, detailing dosages, indications, contraindications, and possible adverse effects. The primary care paediatrician should be continually updated through reports issued during follow-up hospital visits during and after therapy. These reports will include the evolution of the lesion, the response to the drug, any adverse effects, the patient’s general condition, and any indications for further evaluation at national elective centres if surgical or laser treatments are required. At the end of the treatment course, once the hospital record is closed, the patient is returned to the care of the primary care paediatrician, who remains available for any concerns about the recurrence of haemangioma growth, a rare but not impossible event (Figure 3).

## 11. Conclusions

IH is a common condition that can manifest in various patterns, emphasising the need for personalised diagnostic and therapeutic approaches. While many IH cases resolve spontaneously, early detection and accurate risk assessment are crucial to identify those who may undergo serious complications or require immediate intervention. Screening tools like the Infantile Haemangioma Referral Score (IHReS) can help healthcare providers manage patients more efficiently and effectively, leading to better outcomes and reduced risks. Oral propranolol remains the standard treatment for IH cases with potential functional, aesthetic, or life-threatening complications. However, managing IH effectively often requires a multidisciplinary approach involving various specialists, depending on the location and characteristics of the lesion. Ongoing training for paediatricians and dermatologists and digital communication and monitoring tools can improve care quality and ensure consistent treatment pathways. Continued research is essential for advancing our understanding of therapeutic options and long-term treatment effects and refining existing guidelines. Finally, establishing centralised referral networks and shared monitoring systems can help manage complex cases, reduce treatment disparities, and ensure patients receive the most appropriate care.

## Data Availability

No new data were created.
